# Supplementary material for: Marine amphipods (Parhyale hawaiensis) as an alternative feed for the lined seahorse (Hippocampus erectus, Perri 1810): nutritional value and feeding trial
Source: PeerJ. 2021 Oct 19;9:e12288. doi: 10.7717/peerj.12288 (PMC8532987; doi:10.7717/peerj.12288)
Supplement: Supplemental Information 1 — The corresponding PCoA configuration is represented in Fig. 1. [file peerj-09-12288-s001.docx]

| ***Eigenvalues*** |  |  |  |
| --- | --- | --- | --- |
| Axis | Eigenvalue | % | % (cum.) |
| 1 | 1689 | 48 | 48 |
| 2 | 1140 | 32 | 80 |
| 3 | 397 | 11 | 91 |
|  |  |  |  |
| ***Eigenvectors*** |  |  |  |
|  | PCO1 | PCO2 | PCO3 |
| C12:0 | -0.359 | -0.716 | -0.359 |
| C13:0 | 0.137 | -0.549 | -0.501 |
| C14:0 | -0.238 | -0.804 | -0.396 |
| C14:1 | -0.257 | -0.518 | -0.563 |
| C15:0 | 0.728 | 0.003 | -0.322 |
| C16:0 | 0.731 | -0.191 | 0.499 |
| C16:1 | -0.326 | 0.702 | -0.152 |
| C17:0 | 0.645 | 0.652 | 0.062 |
| C17:1 | 0.264 | -0.429 | -0.247 |
| C18:0 | -0.857 | -0.043 | -0.339 |
| C18:1n9c/t | 0.802 | 0.387 | -0.406 |
| C18:2n6c | -0.345 | 0.816 | 0.221 |
| C18:3n3 | -0.763 | 0.454 | -0.157 |
| C20:0 | -0.566 | -0.589 | -0.165 |
| C20:1n9 | -0.547 | -0.689 | -0.240 |
| C20:2 | -0.763 | -0.516 | -0.239 |
| C20:3n6 | 0.815 | -0.418 | -0.178 |
| C20:4n6 | 0.894 | -0.075 | 0.051 |
| C20:5n3 | 0.414 | 0.168 | 0.237 |
| C22:0 | 0.454 | 0.233 | 0.337 |
| C22:1n9 | 0.146 | -0.677 | -0.183 |
| C22:2 | -0.676 | -0.239 | -0.174 |
| C23:0 | 0.574 | -0.534 | -0.334 |
| C24:0 | 0.389 | 0.092 | -0.206 |
| C22:6n3 | -0.826 | -0.182 | 0.240 |
|  |  |  |  |
