# Supplementary material for: Marine amphipods (Parhyale hawaiensis) as an alternative feed for the lined seahorse (Hippocampus erectus, Perri 1810): nutritional value and feeding trial
Source: PeerJ. 2021 Oct 19;9:e12288. doi: 10.7717/peerj.12288 (PMC8532987; doi:10.7717/peerj.12288)
Supplement: Supplemental Information 2 [file peerj-09-12288-s002.docx]

| ***Correlations*** |  |  |  |
| --- | --- | --- | --- |
|  | MDS1 | MDS2 | MDS3 |
| Σ SFA | -0.299 | 0.326 | -0.509 |
| Σ MUFA | -0.422 | -0.141 | 0.465 |
| Σ PUFA | 0.491 | -0.525 | -0.415 |
| n3 HUFA | 0.353 | 0.114 | 0.241 |
| Σ n3 | 0.472 | 0.074 | 0.405 |
| Σ n6 | -0.285 | -0.740 | 0.098 |
| n3/n6 | 0.063 | 0.109 | 0.193 |
| DHA/EPA | 0.107 | -0.073 | -0.249 |
| EPA/ARA | 0.216 | 0.042 | 0.077 |
|  |  |  |  |
