# Supplementary material for: Marine amphipods (Parhyale hawaiensis) as an alternative feed for the lined seahorse (Hippocampus erectus, Perri 1810): nutritional value and feeding trial
Source: PeerJ. 2021 Oct 19;9:e12288. doi: 10.7717/peerj.12288 (PMC8532987; doi:10.7717/peerj.12288)
Supplement: Supplemental Information 3 — The corresponding PCoA is in Fig. 4. [file peerj-09-12288-s003.docx]

| ***Eigenvalues*** |  |  |  |  |
| --- | --- | --- | --- | --- |
| Axis | Eigenvalue | % | % (cum.) |  |
| 1 | 2701 | 52 | 52 |  |
| 2 | 1499 | 29 | 80 |  |
| 3 | 670 | 13 | 93 |  |
| 4 | 247 | 5 | 98 |  |
|  |  |  |  |  |
| ***Eigenvectors*** |  |  |  |  |
|  | PCO1 | PCO2 | PCO3 | PCO4 |
| C12:0 | 0.899 | 0.161 | 0.024 | 0.109 |
| C13:0 | 0.670 | 0.537 | -0.095 | -0.030 |
| C14:0 | 0.893 | 0.307 | -0.086 | 0.035 |
| C14:1 | 0.698 | 0.250 | 0.267 | 0.060 |
| C15:0 | 0.753 | 0.502 | -0.167 | -0.057 |
| C15:1 | -0.113 | 0.208 | 0.068 | -0.332 |
| C16:0 | 0.684 | 0.379 | -0.535 | -0.060 |
| C16:1 | 0.729 | 0.445 | -0.083 | 0.169 |
| C17:0 | -0.355 | 0.515 | 0.022 | -0.216 |
| C17:1 | -0.694 | 0.174 | -0.120 | 0.122 |
| C18:0 | -0.787 | 0.194 | -0.350 | -0.254 |
| C18:1n9c/t | -0.053 | 0.650 | 0.735 | -0.094 |
| C18:2n6c | -0.957 | -0.016 | -0.045 | 0.185 |
| C18:3n6 | -0.680 | -0.382 | -0.072 | 0.379 |
| C18:3n3 | 0.668 | -0.186 | 0.270 | 0.205 |
| C20:0 | -0.250 | 0.260 | -0.110 | 0.157 |
| C20:1n9 | 0.774 | 0.154 | 0.124 | -0.154 |
| C20:2 | 0.580 | -0.225 | 0.256 | -0.044 |
| C20:3n6 | 0.117 | -0.726 | 0.334 | -0.033 |
| C21:0 | -0.265 | 0.502 | 0.047 | -0.283 |
| C20:4n6 | 0.304 | -0.908 | 0.059 | -0.120 |
| C20:3n3 | 0.869 | -0.105 | 0.226 | -0.109 |
| C20:5n3 | 0.602 | -0.637 | 0.229 | 0.102 |
| C22:0 | -0.852 | 0.168 | 0.035 | -0.060 |
| C22:1n9 | -0.084 | -0.018 | -0.006 | 0.040 |
| C22:2 | 0.457 | -0.531 | 0.372 | -0.023 |
| C23:0 | -0.393 | 0.108 | -0.068 | -0.046 |
| C24:0 | -0.764 | 0.025 | 0.051 | -0.099 |
| C22:6n3 | 0.137 | -0.963 | 0.083 | -0.080 |
|  |  |  |  |  |
