# Supplementary material for: Marine amphipods (Parhyale hawaiensis) as an alternative feed for the lined seahorse (Hippocampus erectus, Perri 1810): nutritional value and feeding trial
Source: PeerJ. 2021 Oct 19;9:e12288. doi: 10.7717/peerj.12288 (PMC8532987; doi:10.7717/peerj.12288)
Supplement: Supplemental Information 4 [file peerj-09-12288-s004.docx]

| ***Correlations*** |  |  |  |
| --- | --- | --- | --- |
|  | MDS1 | MDS2 | MDS3 |
| Σ SFA | 0.273 | -0.078 | -0.707 |
| Σ MUFA | 0.359 | 0.106 | 0.586 |
| Σ PUFA | -0.658 | -0.039 | 0.087 |
| n3 HUFA | -0.341 | 0.398 | -0.134 |
| Σ n3 | -0.313 | 0.560 | -0.065 |
| Σ n6 | -0.353 | -0.610 | 0.166 |
| n3/n6 | 0.005 | 0.109 | 0.071 |
| DHA/EPA | -0.143 | -0.299 | -0.218 |
| EPA/ARA | 0.054 | 0.180 | 0.035 |
|  |  |  |  |
